# A Socioecological Assessment of Vulture Abundance and Community Perceptions Before and After Landfill Site Shift in Pokhara, Nepal

**DOI:** 10.1002/ece3.71684

**Published:** 2025-06-26

**Authors:** Binita Timilsina, Mohan Bucha Magar, Sangam Poudel, Dinesh Bhusal, Dipa Gurung, Ankit Bilash Joshi, Yajna Prasad Timilsina

**Affiliations:** ^1^ Institute of Forestry Tribhuvan University Kirtipur Nepal; ^2^ Friends of Nature (FON) Nepal Kathmandu Nepal; ^3^ Department of Forest and Environment Government of Nepal Kathmandu Nepal; ^4^ Bird Conservation Nepal Kathmandu Nepal

**Keywords:** chi‐squared test, perception, scavengers, threats, Wilcoxon‐signed rank test

## Abstract

South Asia is home to nine species of vultures, and Nepal hosts all of them. Remarkably, all these species have also been recorded in Pokhara. This could be attributed to Pokhara's location along bird migration pathways and the year‐round availability of food sources for most of the vulture species, including the landfill. This landfill site has been translocated due to the construction of Pokhara regional international airport. In this context, we aimed to estimate the seasonal abundance of vultures as well as understand the discrepancy in people's perception on vulture conservation before and after the landfill site is shifted to another location. Data were collected using key informant interviews, household surveys, and direct field observations. The collected data were analyzed employing chi‐squared and Wilcoxon‐signed rank tests. The relative abundance of the Egyptian vulture ( *Neophron percnopterus* ) was found to be the highest among observed species. We found an association between people's perception toward vultures and their socioeconomic factors (age, education, and income source). Older people, individuals with higher levels of formal education, and people involved in farming showed greater appreciation for vultures. Our study revealed that the perceived threat of electrocution increased slightly after the landfill site was relocated. Despite the relocation, the old landfill area continues to provide a suitable habitat for vultures, likely due to consistent food availability and the proximity of nesting habitats near forests, cliffs, and rivers. The risk of collisions with airplanes is likely to increase in the future highlighting the need for proactive management and prioritization.

## 1 Introduction

Vultures are important scavengers and critical ecosystem service providers, yet they are among the most threatened bird taxa (Santangeli et al. 2024). Vultures have significant ecological and socioeconomic value. They help to prevent the spread of infectious diseases, reduce odors, and thereby help to maintain a healthy environment by cleaning the carcasses (DNPWC and DoFSC 2023). Besides ecological importance, they also have cultural value. They are thought of as beneficial by many Hindu's who revere them for being carriers of Saturn and believe that Jatayu (vulture) tried to save goddess Sita when Ravana was taking her to his palace after kidnapping her. Vultures also play an important cultural role for Buddhist's in the sky burials (Paudel et al. 2016) in higher elevations of Nepal like Mustang where the traditional Tibetan burial ceremony exposes human corpses to the open air to be eaten by sacred vultures (K. P. Bhusal 2018).

However, in certain communities, people have negative attitudes toward vultures, believing that they bring misfortune and death to their communities (DNPWC and DoFSC 2023). Interactions between vultures and modern civilization has been detrimental to vultures. Negative impacts include electrocution (Magar 2023) or collision with power lines and towers (Gautam and Baral 2013; Joshi 2022), poisoning from nonsteroidal anti‐inflammatory drugs (NSAIDs) like diclofenac, ketoprofen, aceclofenac, nimesulide, cutting down nesting and roost trees, and local food scarcity (Bhusal et al. 2023; Chaudhary et al. 2019; Cook et al. 2024). These ongoing threats should be considered in planning for the long‐term conservation of viable population of vultures in Nepal (Chaudhary et al. 2019; Joshi et al. 2015). Since people are the main reasons for the diminishing status of these huge scavengers, human attitudes may be connected to several chains of both positive and negative effects on these birds. Therefore, people's outlook can have an impact on their ability to survive (Phuyal et al. 2016). Information gaps have a significant influence on conservation efforts for large‐ranging species like vultures (have a range of over 20,000 km^2^) (Gilbert et al. 2006).

There are currently 23 species of vultures worldwide, and Nepal is home to all nine species in the family Accipitridae that are present in the Indian subcontinent (DNPWC and DoFSC 2023). They are classified as critically endangered, endangered, vulnerable, and least concern (Table 1) (DNPWC and DoFSC 2023). The Pokhara valley's landscape is ideal for vultures and is home to all nine species of vulture found in South Asia. Birds are seen circling the valley and foraging on the ground, especially near the city's landfill which is just near the new Pokhara Regional International Airport which opened to flights in 2023 (Dhakal et al. 2022; Joshi 2022). Vulture nesting, feeding, and soaring sites are located within 2 km of the airport (Pokharel 2023). Every year more than 300 vultures are recorded in and around the airport site on International Vulture Awareness Day (Joshi 2022). Species like Egyptian vulture ( *Neophron percnopterus* ) spend their entire life cycles in and around the landfill site. Due to bird air‐strike hazards, a new landfill site was established in Tanahu, but the shift in landfill site has not affected the number of vultures drastically since they return to the vicinity of the airport for breeding and nesting. Due to this, vultures remain a threat to aircraft (Dhakal et al. 2022). Therefore, this research aims to assess the abundance of vultures, identify threats and people's perception on vulture conservation both before and after the shift in landfill site. While research on public attitudes in Pokhara valley was conducted in 2020 (Dhakal et al. 2022), the situation has changed following the inauguration of Pokhara International Airport in 2023. Despite the landfill's relocation; vultures persist in returning to the site due to their nesting habitats on the cliffs and forests opposite the landfill (Gurung 2023).

**TABLE 1:** IUCN status of different vulture species of Nepal.

S. N.	Common name	Scientific name	IUCN status
1	White‐rumped vulture	*Gyps bengalensis*	Critically endangered
2	Indian vulture or long‐billed vulture	*Gyps indicus*	Critically endangered
3	Slender‐billed vulture	*Gyps tenuirostris*	Critically endangered
4	Red‐headed vulture	*Sarcogyps calvus*	Critically endangered
5	Egyptian vulture	*Neophron percnopterus*	Endangered
6	Bearded vulture	*Gypaetus barbatus*	Vulnerable
7	Cinereous vulture	*Aegypius monachus*	Vulnerable
8	Himalayan vulture	*Gyps himalayensis*	Vulnerable
9	Griffon vulture	*Gyps fulvus*	Least concern

*Note:* Vulture Conservation Action Plan (2023–2027), Department of National Park and Wildlife Conservation (2023).

In this regard, this research provides current insights into the abundance of vultures; threats posed by the airport and the evolving perspectives of local communities in response to the landfill's shift. The findings will serve as a foundational reference for future studies, balancing the imperatives of environmental conservation and urban development. Furthermore, the study will contribute to revising and updating the vulture conservation action plan, underscoring the essential role of vultures in urban ecosystems like Pokhara, where rapid urbanization necessitates thoughtful ecological strategies.

## 2 Materials and Methods

### 2.1 Study Area

**FIGURE 1:**
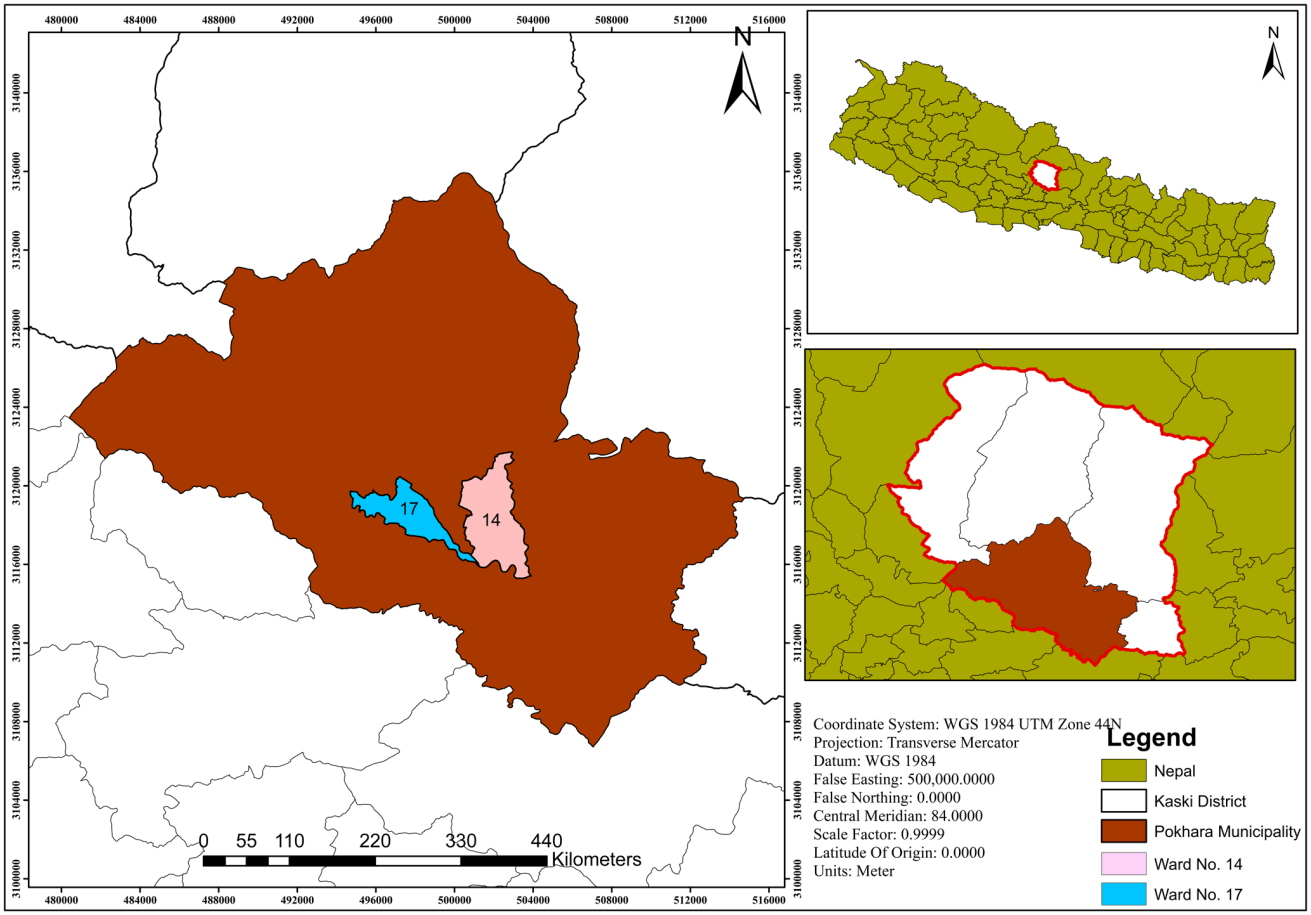
Map of the study area showing Nepal with Kaski district highlighted, and the location of Pokhara metropolitan city. The enlarged section displays wards 14 and 17, located on opposite sides of the former landfill site.

Pokhara city lies within Kaski district (28°18′19.08″ N and 84°04′37.20″ E) of Gandaki province. Its significant altitudinal variation supports a diverse climate, ranging from subtropical to tundra, with a temperature range from 7°C to 31°C (Kharel and Basnet 2021). The city features a rich blend of diverse communities, including Brahmins, Kshetris, Thakalis, Newars, Gurungs, and Magars (Bhandari 2024). This study was conducted in wards near the old landfill site (Ward nos. 14 and 17) of Pokhara Metropolitan city (Figure 1). The two wards lie on either side of Seti River. The habitat features abundant mature Simal trees ( *Bombax ceiba* ), rugged rocky cliffs, and dispersed human settlements, creating ideal habitats for various highland species, such as Himalayan vulture ( *Gyps himalayensis* ), White‐rumped vulture ( *Gyps bengalensis* ), Red‐headed vulture ( *Sarcogyps calvus* ), Slender‐billed vulture ( *Gyps tenuirostris* ), Cinereous vulture ( *Aegypius monachus* ), and Bearded vulture ( *Gypaetus barbatus* ) (Baral 2021).

### 2.2 Data Collection

#### 2.2.1 Key Informant Survey

Altogether 10 key informants were selected based on their expertise, institutional roles, and local knowledge. They were consulted to extract reliable information regarding the presence of vultures and their threats in the periphery of study site. These informants include members of government, NGO officials, the management section head of the airport, bird experts, and other key‐stakeholders Information received from key informants was used to cross‐check the household survey (described below) results that provided contextual information for a better overview and interpretation.

#### 2.2.2 House‐Hold Survey

Semi‐structured questionnaire surveys were conducted among local people of a range of ages to collect information regarding threats to vultures both before and after shifting the landfill site. The sampling universe was 5700 households in the study area, and 200 (3.5%) were randomly selected for surveys. The sample included 103 respondents from Ward No. 17 (located farther from the landfill site) and 93 respondents from Ward No. 14 (closer to the landfill site). We aimed to understand inhabitants' perception about vultures, carcass disposal practices, and livestock holdings. The sample households were selected based on Slovin Formula with 10% of marginal error (Glen 2024) which is given by

$$n=N/\left(1+N\;e^{2}\right),$$

where *n* is required sample size, *N* is the total number of households in the study area, and *e* is the marginal error.

Socioeconomic factors were grouped as needed for chi‐squared test. Major sources of income were classified as income from remittances, salary or wage based, and agricultural and livestock ownership. Similarly, education levels were categorized as no formal education, primary, secondary, and higher secondary education. However, age was considered a continuous variable, using actual ages reported by respondents. The threat score was given by the respondents for each threat from 1 to 3 with 3 being the highest threat.

#### 2.2.3 Vulture Survey

To assess the number of vultures present in the area, vultures were counted along the trails used by the local people. Besides counting birds, their activity and the major vegetation type in the surrounding area were noted. A total of seven transects were surveyed with an average length of 1 km. A minimum of three surveys were conducted, and in some months up to five; from November to May on days with good visibility at times when vultures were most active (08:00–12:00 and 15:00–17:00) (Karki et al. 2019).

### 2.3 Data Analysis

#### 2.3.1 Seasonal Abundance

The relative abundance (RA) of each species observed was determined using the equation:

$$\mathrm{RA}\;\left(\%\right)=\text{34𝑛/34𝑛𝑁}\times 100,$$

where *n* is the number of individuals of particular recorded species and *N* is the total number of individuals of recorded species.

#### 2.3.2 People's Perception and Threats

The data derived from questionnaires were analyzed using SPSS Version 27 (George and Mallery 2019). The descriptive statistics, mean, frequency, minimum, and maximum value were calculated and then logically interpreted along with simple tables, charts, and graphs. The nonparametric chi‐squared test (*χ* ^2^) was used to determine the association between various socio‐economic variables (age, gender, education, years of stay, distance from landfill site, etc.) and the perception of people towards vultures. Similarly, the nonparametric Wilcoxon‐signed rank test was performed to examine threats. The threats (human persecution, NSAIDs, poisoning, habitat loss, collision with airplanes, and transmission lines) were ranked using the Likert scale (Kankam and Abukari 2020) as high, medium, and low both before and after the shift in the landfill site.

## 3 Results

### 3.1 Seasonal Abundance of Vultures

Egyptian vultures ( *Neophron percnopterus* ) were by far the most abundant of the seven species, and they were over twice as common in winter as in spring. They comprised more than 70% of all vultures seen in winter and over 60% in spring. Other species seen in relatively high numbers were White‐rumped vulture ( *Gyps bengalensis* ), Griffon vulture ( *Gyps fulvus* ), and Himalayan vulture ( *Gyps himalayensis* ), whereas relatively fewer individuals were recorded for the other species. All species were more common in winter than in spring, with the disparity being lower for White‐rumped vulture ( *Gyps bengalensis* ), Himalayan vulture ( *Gyps himalayensis* ), and Slender‐billed vulture ( *Gyps tenuirostris* ) than for the others.

**FIGURE 2:**
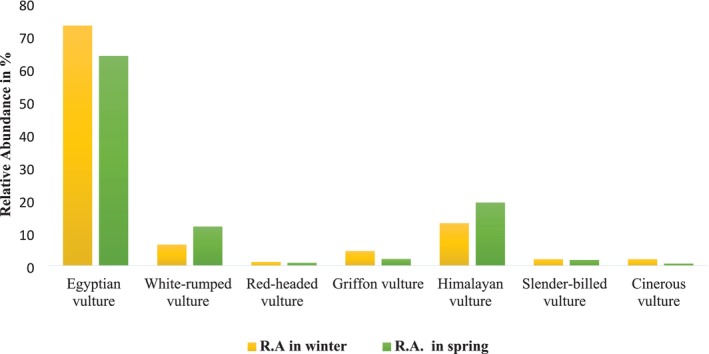
Seasonal abundance of vultures in and around the landfill site of Kaski. The *y*‐axis represents the relative abundance (%) of each vulture species, whereas the *x*‐axis compares their abundance across two seasons: Winter and Spring.

The relative abundance of Egyptian vulture ( *Neophron percnopterus* ) was found to be highest (73% in winter and 64% in spring), whereas the least abundant was Red‐headed vulture ( *Sarcogyps calvus* ) (1.1% in winter and 0.84% in spring). The relative abundance of Cinerous vulture ( *Aegypius monachus* ) (2% in winter and 0.5% in spring) and Griffon vulture ( *Gyps fulvus* ) (4.5% in winter and 2% in spring) was higher in the winter season because they are winter migratory birds. However, the abundance of White‐rumped vulture ( *Gyps bengalensis* ) (12% in spring and 6% in winter) and Himalayan vulture ( *Gyps himalayensis* ) (19% in spring and 12% in winter) was higher in spring than in winter (Figure 2).

Throughout the 6‐month study period, landfill area served as habitats for various avian species associated with vultures such as Steppe eagle ( *Aquila nipalensis* ), Black kite ( *Milvus migrans* ), Jungle crow ( *Corvus macrorhynchos* ), Common myna ( *Acridotheres tristis* ), Cattle egret ( *Bubulcus ibis* ), Red‐wattled lapwing ( *Vanellus indicus* ), Shikra ( *Accipiter badius* ), Common kestrel ( *Falco tinnunculus* ), Red‐vented bulbul ( *Pycnonotus cafer* ), and Black drongo ( *Dicrurus macrocercus* ).

### 3.2 People's Perception Toward Vulture Conservation

**FIGURE 3:**
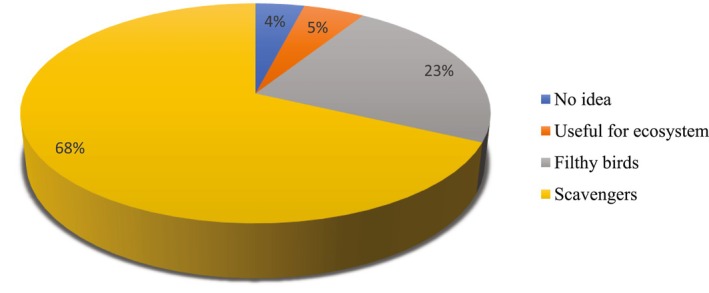
Respondent's view toward vulture. The chart illustrates the range of perceptions among participants, including those who considered vultures as useful for the ecosystem, viewed them as scavengers, regarded them as filthy birds, or had no clear idea about their role.

Out of 200 respondents, 136 respondents (68%) believed that the vultures are the natural scavengers having a crucial role in ecosystems for maintaining a healthy, hygienic, and balanced environment. Very few (4%) households were unaware of the ecological importance of vultures (Figure 3).

The perception of vultures among respondents, especially in terms of their usefulness as scavengers and their role in preserving ecosystem health, was influenced by factors such as education, age, gender, years of residence, and primary income source. A nonparametric chi‐squared test revealed significant associations between perceptions of vultures and age, education level, and major source of income (Table 2).

**TABLE 2:** People's perception toward conservation of vultures with different socioeconomic variables.

Variables	*χ* ^2^ value	df (degree of freedom)	*p*
Age	24.373*	6	< 0.001
Education level	24.566*	9	< 0.01
Major source of income	17.132*	6	< 0.01
Years of stay	7.605	6	0.269
Gender	2.258	3	0.521

* People's perception toward conservation of vulture is statistically significant with the variables such as age, education level, and source of income.

Older people seemed more inclined to recognize and appreciate the ecological benefits that vultures offer. This may be because of their greater exposure to traditional ecological knowledge and direct experiences with the advantages vultures offer. Individuals with higher levels of formal education exhibited a greater recognition of the ecological significance of vultures, indicating that education improves environmental awareness. Additionally, people involved in farming and livestock‐related livelihoods showed greater appreciation for vultures, probably because they can directly get benefits they provide‐ the natural removal of animal remains and the avoidance of disease outbreaks. On the other hand, there was not any association between people's perception towards vulture and gender, and years of stay. This suggests that opinions on vultures were fairly uniform among these demographic categories.

**FIGURE 4:**
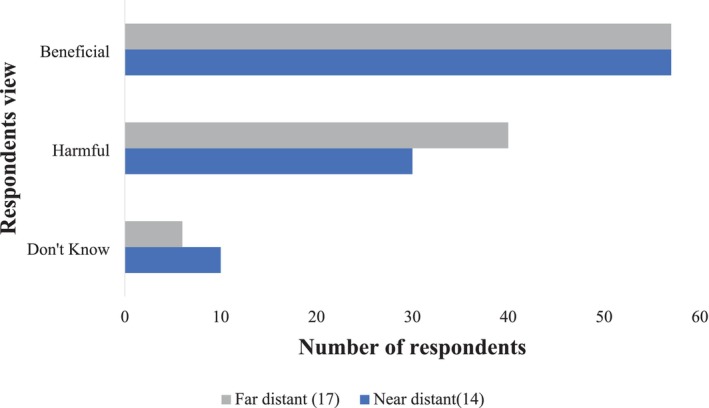
Views of vultures held by respondents residing at near distance (Ward 14) and far distance (Ward 17) from the landfill location.

Interestingly, the number of respondents considering vultures as beneficial species was equal in number from both far and near distant category. However, the respondents believing vultures as harmful species were higher in areas far from landfill site (39% in Ward No. 17) than that in the areas near to the landfill site. According to locals, the lack of awareness initiatives in remote areas likely contributed to the negative perception of vultures. No organizations or groups had visited these areas to educate people about the ecological importance of vultures. Moreover, residents often confused vultures with eagles, which are known to harm livestock and hence thought vultures as harmful species. Additionally, some locals blamed vultures for releasing white, fluid feces in agricultural fields, which they perceived as making the land dirty (Figure 4).

### 3.3 Threats Before and After Shift in Landfill Site

When questioned about the changes in the number of vultures before and after shifting the landfill site, 155 out of 200 respondents reported noticing a difference, while the remaining 45 did not observe any changes due to being unaware of the landfill's relocation. Among those who noticed changes, 84.6% (131) respondents observed a significantly higher number of vultures before the landfill site was shifted, followed by a drastic decline afterward. In contrast, 7.7% respondents reported fewer vultures before the shift, 2.5% noticed no change in numbers, and 5.2% were uncertain about any increase or decrease. Electrocution and collisions with transmission lines were identified as the most significant conservation threats following the relocation. The primary reason for the landfill site's shift was the establishment of the Pokhara International Airport, located just 2 km away, threatening the habitat and food access of vultures. Additionally, many respondents reported incidents of bird collisions with airplanes, particularly involving raptors, further exacerbating the risks to vulture populations in the area.

**FIGURE 5:**
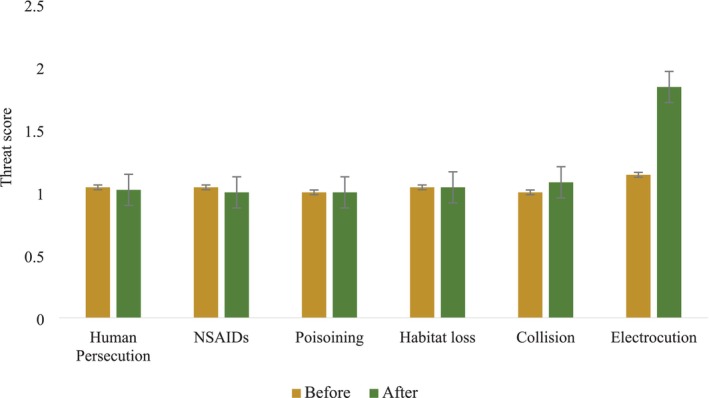
Perceived threats to vultures before and after shifting landfill site. *X*‐axis represent various identified threats to vultures and *y*‐axis represent average threat score given by respondents.

The Wilcoxon‐signed rank test revealed that threat scores for human persecution (*z* = −2.00, *p* = 0.046), NSAIDs (*z* = −2.00, *p* = 0.046), Poisoning (*z* = 0, *p* = 1), and habitat loss (*z* = 0, *p* = 1) showed no significant differences before and after shifting landfill site. The mean threat scores before and after the shift were as follows: human persecution (1.04 and 1.02), NSAIDs (1.04 and 1.00), poisoning (1.00 and 1.00), and habitat loss (1.04 and 1.04), in which all these values are deviated toward 1, indicating a low level of perceived threat (Figure 5).

In contrast, the threat scores for electrocution (*z* = −9.725, *p <* 0.001) and collisions with airplanes (*z* = −3.314, *p <* 0.001) were found to be significant. The mean threat scores for electrocution increased from 1.14 to 1.84, indicating an increase in perceived risk after the landfill relocation. In the case of airplane collisions, although the mean score increased slightly from 1.00 to 1.08, this small but statistically significant change likely reflects heightened awareness. This may be because of high foraging, nesting, and roosting activities of vultures in close proximity to the new regional international airport. The overlap with airplane routes, particularly during takeoff and landing, may significantly increase the risk of vulture‐airplane collisions in the future.

## 4 Discussion

### 4.1 Seasonal Abundance

We found only seven of the nine species of vultures found in Nepal. In our study area, although Bearded vulture ( *Gypaetus barbatus* ) was previously recorded, there is no record of Indian vulture ( *Gyps indicus* ) in the areas (Dhakal et al. 2022; Shah et al. 2019). The most abundant species (Egyptian vultures) in our study are the same as those in a previous study (Dhakal et al. 2022). One of the reasons Egyptian vulture ( *Neophron percnopterus* ) was the most abundant species at the landfill in our study and that of Ghimire et al. (2020) is probably because of its inability to compete with larger vultures around large carcasses elsewhere. Our findings also correspond with the study by Saran and Purohit (2014) in Jodhpur, India that found the abundance of Egyptian vulture ( *Neophron percnopterus* ) was highest during the winter season (November–February) suggesting this timeframe is ideal for population estimation. The Egyptian vulture's proximity to towns is likely due to its habit of feeding at garbage dumps and on human or livestock waste (Ghimire et al. 2020). Similarly, the Himalayan vulture ( *Gyps himalayensis* ) and Griffon vulture ( *Gyps fulvus* ) have similar appearances, leading many locals to mistakenly identify them as the same species, resulting in the observation of these two species in significant numbers after Egyptian vulture ( *Neophron percnopterus* ).

Landfill sites are known as hotspots for scavengers (Arnold et al. 2021; DeCandido et al. 2012) and at our site, factors that attracted vultures were the abundance of mature *Bombax ceiba* trees along the forest periphery for nesting and roosting and easy access to food and water provided by the dumping site and the nearby Seti River.

### 4.2 People's Perception on Vulture Conservation

In Ghimire et al. (2020), conservation attitudes were found to be influenced by socioeconomic variables such as gender, ethnicity, education, occupation, years of residence, and age. Similarly, our study confirmed that individuals of varying ages, educational backgrounds, and income sources had differing perceptions of vultures. A study in Spain revealed that a positive attitude toward vultures among farmers who were familiar with vultures through personal experience and had ecological knowledgesupports our study outcomes as well (Morales‐Reyes et al. 2018). According to Manqele et al. (2023), respondents had a good awareness of vultures and acknowledged the advantages of their presence in the community, which is comparable with our observations. Findings from the Newmont Akyem Enclave (NAE) in the eastern part of Ghana showed that support for vulture conservation was stronger among individuals who opposed their persecution (Kankam and Abukari 2020). This pattern is in line with prior studies in Ghana reflecting vultures as beneficial birds to urban and rural ecosystems (Boakye et al. 2019; Deikumah 2020). Providing the same reasons, our study revealed that more than 50% of the respondents perceived vultures to be useful. However, when asked about conservation efforts for vultures, most respondents emphasized the need to protect their natural habitat, particularly by preserving the simal ( *Bombax ceiba* ) trees in the nearby forest. They also suggested educating local communities about the ecological importance of vultures and the environmental consequences of their absence. This knowledge is most likely influenced by formal education and the region's strong tourist industry.

### 4.3 Threats to Vulture After Shifting Landfill Site

When comparing the seriousness of threats before and after the landfill site shift, factors such as human persecution, NSAIDs, poisoning, and habitat loss did not exhibit significant changes. However, electrocution with power lines are perceived as the primary threats, followed by collisions with airplanes, particularly due to the newly constructed international airport in Pokhara which is contrary to another study which identified habitat loss from urbanization to be primary threat in Pokhara valley (Dhakal et al. 2022). In Karki et al. (2019), of a total of 200 respondents, 45% attributed the decline in vulture numbers to the use of veterinary drugs like diclofenac, 37% to food shortages, 12% to habitat destruction, 5% to pesticides, and 1% to electrocution, persecution, poisoning, and herbicides; which differ from our findings. While the Vulture Conservation Foundation, V. C. F (2024) recorded the electrocution of two Griffon Vultures on medium‐voltage lines in Cyprus, our study observed no such cases, likely due to low abundance of this species in the study area. We found a dead White‐rumped vulture under transmission line in landfill area. Vultures are large birds and they are often electrocuted on power lines and such accidents are often observed in winter when poor visibility increases as they migrate to new areas. Other causes include human persecution, such as killing vulture during carcasses feeding, cutting down brood trees and catching chicks from their nests that is consistent with our findings (Chaudhary et al. 2019). As studied by Ogada et al. (2012), vultures face numerous threats due to multiple anthropogenic factors including poisoning, loss of habitat, policy changes, and infrastructure fatalities. In the context of our study, anthropogenic disturbances caused by the construction of an international airport could affect vulture feeding grounds and roosting around the site. While most earlier studies highlighted diclofenac use as a critical threat to vultures (Gautam and Baral 2013), local respondents in our study reported no current use of diclofenac in the area, likely due to its ban on import and manufacture in Nepal. In addition to this, excessive use of chemicals and pesticides in agriculture, lack of conservation awareness, and hunting are depleting the area's vulture population (Gurung 2023).

## 5 Conclusion

Seasonal abundance and richness of vultures may vary depending on weather pattern and migration timing. These records could help boost vulture tourism as well as applying more preventive measures in international airport near landfill site to reduce chances of collision. According to local respondents, electrocution was perceived as the primary threat to vultures in the area. While we did not empirically test this threat, we recommend using insulators in poles which could help save vultures and other bird species. Although most people perceive vultures as useful, about one third of the respondents take them as harmful which shows the lack of awareness among people even in Pokhara valley which needs to be addressed. Active involvement of local communities across the vultures' range is imperative to the success of such programs. Since these findings are based on community perceptions and may not fully capture the actual ecological dynamics or all existing threats to vulture populations. The statistical analyses were limited to descriptive and nonparametric tests that may limit the ability to detect interaction effects between socioeconomic variables and future work could build on this with multivariate techniques. The use of local ecological knowledge can offer important insights into community‐driven conservation priorities, especially in data‐deficient contexts. However, we recommend future studies to be focused on empirical studies finding bird species that are prone to electrocution and collision along with the status of electrocution and collision records in Pokhara valley.

## Author Contributions

**Binita Timilsina:** conceptualization (lead), data curation (lead), formal analysis (lead), funding acquisition (lead), investigation (equal), methodology (lead), resources (lead), software (supporting), writing – original draft (lead), writing – review and editing (equal). **Mohan Bucha Magar:** investigation (supporting), methodology (equal), validation (supporting). **Sangam Poudel:** conceptualization (supporting), investigation (supporting), writing – original draft (supporting), writing – review and editing (equal). **Dinesh Bhusal:** data curation (supporting), investigation (supporting), methodology (supporting), writing – original draft (supporting), writing – review and editing (equal). **Dipa Gurung:** data curation (supporting), investigation (supporting), writing – original draft (supporting), writing – review and editing (equal). **Ankit Bilash Joshi:** conceptualization (equal), data curation (equal), formal analysis (equal), methodology (equal), supervision (lead), writing – original draft (equal), writing – review and editing (equal). **Yajna Prasad Timilsina:** conceptualization (equal), formal analysis (lead), methodology (equal), software (lead), supervision (lead), validation (equal), writing – review and editing (equal).

## Ethics Statement

Ethical approval was granted by Institute of Forestry, Pokhara Campus to conduct the research. Informed consent was obtained from all participants while carrying out questionnaire survey and all personally identifiable information was anonymized to ensure confidentiality and privacy.

## Conflicts of Interest

The authors declare no conflicts of interest.

## Data Availability

Datasets are available from the corresponding author upon request.
